# Are mental dysfunctions autonomous from brain dysfunctions? A perspective from the personal/subpersonal distinction

**DOI:** 10.1007/s44192-024-00117-x

**Published:** 2024-12-02

**Authors:** Marko Jurjako

**Affiliations:** Department of Philosophy and Division of Cognitive Sciences, Faculty of Humanities and Social Sciences, University of Rijeka, Sveucilisna avenija 4, 51000 Rijeka, Croatia

**Keywords:** Mental dysfunction, Brain dysfunction, The interface problem, Personal/subpersonal, Neurocognitive and computational psychiatry

## Abstract

Despite many authors in psychiatry endorsing a naturalist view of the mind, many still consider that mental dysfunctions cannot be reduced to brain dysfunctions. This paper investigates the main reasons for this view. Some arguments rely on the analogy that the mind is like software while the brain is like hardware. The analogy suggests that just as software can malfunction independently of hardware malfunctions, similarly the mind can malfunction independently of any brain malfunction. This view has been critically examined in recent literature. However, other less discussed reasons suggest that mental dysfunctions cannot be reduced to brain dysfunctions because mental dysfunctions are appropriately ascribed at the level of intentional mental states, while brain dysfunctions are solely related to abnormalities in anatomy and physiological processes. This paper questions why such a view would be upheld. The discussion is framed within the interface problem in the philosophy of cognitive science, which concerns the relationship between personal and subpersonal levels of explanation. The paper examines the view that an autonomist perspective on the personal/subpersonal distinction could justify the separation of mental dysfunctions, described in intentional terms, from brain dysfunctions, described in anatomical or physiological terms. Ultimately, the paper argues that the autonomist view cannot be upheld in psychiatry and, consequently, does not provide a principled justification for rejecting the reduction of mental dysfunctions to brain dysfunctions.

## Introduction

In theoretical and practical approaches to psychiatry, there is an ongoing debate about whether mental disorders can and should be reduced to brain disorders. While some, such as proponents of the Research Domain Criteria (RDoC) advanced by the American National Institute of Mental Health, argue that for practical and research purposes, mental disorders should be reconceived as brain disorders [1–4], many still contend that mental disorders are explanatorily autonomous and cannot be reduced to brain disorders [5–8]. For instance, the idea would be that a person could suffer from depression caused by dysfunctional thought processes, involving entrenched negative thought patterns that are implemented in a perfectly functioning brain, i.e., a brain that doesn’t exhibit any form of dysfunction. This situation is peculiar because, at the same time, many in the philosophy of mind and its applications to practical disciplines, such as psychiatry, accept some form of naturalism about the mind [9]. The idea is that the mind reduces to the brain and other relevant bodily processes, or at least supervenes on them [6, 10–12]. If the mind essentially depends on brain processes, then why think that mental disorders can be autonomous from brain disorders? Moreover, if there are principled reasons to consider mental disorders as autonomous from brain disorders, this might suggest that initiatives like the RDoC—which aim to reconceptualize mental disorders as disorders in neural and other bodily processes—rest on a conceptual confusion. These issues prompt the discussion in the present paper.

Often, such autonomist views are justified based on the computer analogy, according to which minds are like software and brains are like hardware [8, 13–15]. The idea here is that, just as we have a clear understanding of how software can malfunction even when implemented on fully functional hardware, we can similarly understand how mental processes might be dysfunctional while being realized in a perfectly functioning brain. If the mind is analogous to software and the brain to the implementing hardware, this analogy would clarify how mental dysfunctions could occur independently of any brain dysfunction [15].

However, there are also less discussed arguments that don’t directly rely on the computer analogy. For instance, some claim that what is characteristic about mental disorders is that they are defined by dysfunctions in intentional processes and consciousness, while brain disorders are defined by abnormalities or dysfunctions in anatomical structure and/or physiological processes [5, 6, 8].^1^ According to these views, if mental dysfunctions cannot be reduced to brain dysfunctions identified solely in non-intentional terms, then these mental dysfunctions would be autonomous from brain dysfunction. Consequently, mental disorders would also be autonomous from brain disorders. However, it is often assumed, rather than argued, that brain dysfunctions should be characterized entirely in non-intentional terms. This paper focuses on evaluating this type of argument for the claim that mental dysfunctions are autonomous and irreducible to brain dysfunctions.

To make progress on this issue, this paper will approach the topic by reflecting on the interface problem from philosophy of psychology and how it applies to theoretical issues in psychiatry (see, also [16]). The interface problem is the problem of understanding the relation between the personal and subpersonal levels of explanation [21]. According to autonomist approaches to the personal/subpersonal distinction, it makes sense only to ascribe psychological predicates, like beliefs and desires, to whole agents described at the personal level [22, 23]. That is the level at which we explain people’s behavior in terms of their goals, reasons, rational capacities, and conscious experiences. In contrast, the subpersonal level pertains to parts of agents that fall outside of conscious experiences and are normally characterized in causal/nomological or mechanistic explanations. Consequently, according to autonomist views, ascribing intentionality to subpersonal processes would involve some kind of conceptual confusion or illegitimate form of explanation [22, 24]. Thus, accepting the autonomist view would vindicate the claim that brain dysfunctions, as attributes of parts of agents, cannot be described in intentional terms.

However, using the autonomist view of the personal/subpersonal distinction to argue for autonomous mental dysfunction/disorder merely shifts the problem to why we should accept this view of the relation between the personal and the subpersonal.

In the remainder of this paper, we will explore the prospects and viability of employing the autonomist view within contemporary theoretical and empirical approaches to psychiatry. To frame the discussion, this paper will focus on Jerome Wakefield’s [8] work, in which he purports to demonstrate an actual case of a mental dysfunction without a corresponding brain dysfunction. The purpose of rehashing his argument is to extract the basic elements for constructing the argument supporting the thesis that mental dysfunctions do not entail brain dysfunctions.

The paper is structured as follows. Section 2 provides a review of Wakefield’s [8] arguments for distinguishing mental dysfunction from brain dysfunction. Section 3 discusses some preliminary reasons, based on the software/hardware analogy and the multiple realizability of the mental, for thinking that brain dysfunctions shouldn’t be described in intentional terms. Section 4 introduces a potential justification, based on the autonomist view of personal and subpersonal levels, for why brain dysfunctions shouldn’t be described in intentional terms. Section 5 critically examines this view and argues that it doesn’t adequately reflect psychiatric theory and practice. Section 6 concludes the discussion.

## Can there be mental dysfunction without brain dysfunction?

Over the years, Wakefield [19, 25] has become one of the most influential proponents of a hybrid account of mental disorders [16]. According to Wakefield, a condition is classified as a disorder if it causes harm to a person, and this harm results from a dysfunctional bodily or psychological mechanism. The view is termed “hybrid” because the harm condition is determined by social values, while the dysfunction condition is intended to be objective, involving a failure in a mechanism that has been shaped by natural selection.

For our purposes, the important aspect of this view is the dysfunction condition. The question is whether a mental disorder, as a consequence of a dysfunctional psychological mechanism, also implies that there is some part of the brain that is dysfunctional. Wakefield [8, 26], answers in the negative. To see why, let us examine his reasoning about this issue.^2^

### Dysfunctional mental mechanisms in a functional brain? The case of the fox and the gosling

As many in the literature do, Wakefield accepts the physicalist view of the mind, according to which mental states are instantiated or realized in brain states [11, 28]. It would follow from this that dysfunctional mental states are also realized and somehow “reside” in brain states. However, Wakefield [8] notes that while mental dysfunctions are necessarily realized in the brain, this does not mean that any of the brain states are necessarily *dysfunctional*.

To support this view, Wakefield [8, 10] attempts to provide an example of a malfunctioning psychological mechanism that does not involve any underlying brain dysfunction. In this regard, he uses the case of the imprinting mechanism found in many species of birds. The imprinting mechanism involves a set of neural assemblies whose role is to create a representation of some feature of what they are exposed to upon hatching. For instance, goslings exposed to a moving stimulus for several minutes after hatching will typically develop affiliative attachments to this moving stimulus [34]. Typically, the first thing they see is their mother, and they respond with behaviors that include closely following her. The imprinting mechanism is adaptive because it allows for rapid learning, leading to attachment to a figure that will ensure their protection and provide food. Thus, imprinting in normal circumstances enables survival. Wakefield plausibly suggests that, at higher levels of psychological description, the function of the imprinting system could be understood as involving the representation of the mother goose, as this effect is likely what the mechanism was selected (for further discussion of mental representation, see [28]).

Now, following Wakefield [8], let us imagine that the first thing a gosling sees upon hatching is a fox. The gosling’s neural assemblies register the fox as its mother, and it starts to follow the fox around. This eventually leads to the premature death of the gosling. Wakefield contends that, in such a case, the imprinting mechanism would malfunction because, instead of discharging its function by targeting the mother, its misfiring leads to attachment to the fox and premature death.

It is debatable whether this would be a genuine case of a mechanism that is dysfunctional or a mechanism that functions properly but cannot execute its proper function due to an unfriendly environment [9, 28]. For instance, if a person ends up in space without a spacesuit, they would not be able to breathe. However, the explanation for this would not involve a malfunction in the respiratory system; rather, the environment is such that the respiratory system cannot execute its function properly. Wakefield claims that the gosling case is different because imprinting on a wrong target leads to disruptions in the developmental processes. Indeed, Wakefield [8] claims that imprinting on the wrong target leads to irreversible changes in developmental processes, causing failures in various important functions. The downstream effects of misimprinting on internal processes indicate that the main problem is internal dysfunction, rather than a mismatch with the external environment where the imprinting mechanism cannot function properly. According to Wakefield’s reasoning, the primary disanalogy with the failing respiratory system in space is that wrong imprinting is irreversible and depends on a crucial moment in the developmental stage of goslings.

Whether Wakefield provides a completely compelling example is less important for the present discussion.^3^ For the sake of argument, let us suppose that he offers a credible example of a psychological dysfunction. What is more important is why Wakefield believes this is a case of a dysfunctional psychological mechanism that does not involve a dysfunctional brain process. His claim is that although the gosling’s imprinting process is dysfunctional because the representation that was formed was not of the mother, at the level of the neural assemblies that realize the imprint function, everything is functioning as designed. How can this be the case if mental functions have been shaped by natural selection via the design of the brain?

### Intentional mental content vs. structural brain dysfunction

Wakefield believes this can be so because what often characterizes mental processes is their intentional content and their role in explaining other intentional phenomena. In contrast, he construes brain dysfunctions as abnormalities in the structural or physiological properties of the brain. He explains this as follows:

“The answer is that the dysfunction at the psychological level concerns intentional representational content, not neurophysiology; and it concerns not whether the psychological process of imprinting took place successfully (it did), but what it is that the gosling imprinted on, a question outside of neurophysiology. The image in the brain refers to a passing fox, not to the mother, and thus involves the failure of a higher level of function of the imprinting mechanism, namely, that the gosling imprint on the mother. One can identify what has gone wrong only by going beyond brain descriptions and referring to meanings (i.e., what the gosling’s brain-stored image in fact represents). (…) So, we have here an example of a psychological dysfunction that is not a brain dysfunction because it cannot be described in sheerly brain-physiological terms.” [6–8].

Wakefield’s claim seems to be that, at least in this case, psychological or mental dysfunctions are specifically those that can be characterized in terms of disturbances in intentional processes, at the level of informational content or meaning. In contrast, proper brain dysfunctions are those that can be characterized in non-intentional terms, referring only to brain structures and physiological processes. Indeed, in more general terms, Wakefield defines brain disorders as follows:

“A brain disorder is a harmful dysfunction of brain mechanisms in which the function that is failing to be performed can be fully specified in brain-anatomical and brain-physiological terms without any essential reference to the psychological/mental level of description involving the intentional field or conscious experiences.” [4, 8].

The view suggests that brain dysfunctions are disturbances in the neural mechanisms that can be described without presupposing the application of psychological predicates. In other words, brain dysfunctions can be described in terms of abnormal anatomical structures or disturbances in physiological processes. In contrast, an autonomous mental disorder would involve a dysfunction in mental mechanisms that can be fully specified in terms of disturbances in intentional processes or conscious experiences [35]. Indeed, if a mental disorder involves a dysfunction in intentional processes, but brain disorders cannot involve processes described in those terms, it follows that mental dysfunctions can exist without brain dysfunctions, and, consequently, mental disorders can exist without brain disorders.^4^

## Can brain dysfunctions be intentional?

Now we may ask, what justifies accepting this view that requires non-intentionality of brain dysfunctions? Wakefield [8] doesn’t offer a clear justification for it, but treats it as a commonly accepted distinction between mental and somatic states. Others have adopted similar views [5, 6, 36, cf. 37]. For instance, in a different context, Denny Borsboom et al. [5] argue against explanatory biological reductionism in psychiatry by claiming that many psychiatric disorders involve intentionally characterized phenomena, which precludes their being successfully reduced to the biological level because, at that level, the phenomena presumably are not intentionally characterized. But, as in the case of Wakefield, it remains unclear why the purported brain phenomena should be characterized in non-intentional terms.

In what follows, we will briefly discuss two potential justifications for such a view—the computer analogy and multiple realization of the mental—and outline their limitations in this context. Setting these arguments aside will allow us to explore in Sect. 4 a neglected perspective on this issue that is based on the distinction between personal and subpersonal views of the mind/brain.

### The autonomy of mental dysfunction: The software/hardware analogy

Many have argued that mental dysfunctions can easily be conceived as autonomous from brain dysfunctions when we consider the computer metaphor, where the mind is akin to complex software and the brain to the hardware that implements it [8, 11, 13, 14]. The standard idea is that software can malfunction even if the hardware in which it is realized is functioning as designed. This is possible because hardware has a separate design specification from software, and vice versa. Usually, hardware is designed as a universal computer that can implement different types of software. In contrast, software is designed for specific purposes, goals, or functions it is supposed to accomplish. Given that they are designed separately for their own purposes, it is clear that glitches in software can be independently identifiable and therefore considered dysfunctional without considering anything about the implementing hardware as dysfunctional. By analogy, given that the mental is supposed to stand in the same relation to the brain as software does to hardware, it follows that mental dysfunction does not necessitate brain dysfunction.

However, there are at least two reasons why drawing an analogy between mind/software and brain/hardware does not justify characterizing brain dysfunctions solely in non-intentionalist terms. The first reason is that, in general, the analogy fails to justify a distinction between mental and brain dysfunctions. Harriet Fagerberg [15] recently argued forcefully that the analogy is not compelling because the mind does not have a functional profile independent from the functional profile of the brain in the way that software is generally functionally independent from the functional characterizations of hardware [11]. This is especially the case if the notion of function/dysfunction is understood etiologically [19, 29–31].^5^ From a naturalistic perspective, the mind is simply a set of capacities enabled by the brain. These capacities were shaped by biological evolution, influenced by the natural selection of the brain and other bodily features [28]. Consequently, there is no separate evolutionary history for mental capacities that is not associated with the evolution of the brain and other parts that embody the mind. In other words, the brain is not independently “designed” by natural selection for mental processes to be separately “installed”. Rather, mental functions emerge as effects of neural processes that were shaped, among other things, by natural selection. Thus, the design profile of the mind is constitutively related to the design profile of the brain. Consequently, according to Fagerberg [15], if there is dysfunction in the mind, such as a gosling’s imprinting system failing to encode a representation of its mother, this dysfunction would necessarily indicate that something in the brain is not functioning as it should.^6^

Second, relying on the software/hardware analogy cannot justify Wakefield’s view of brain dysfunction being necessarily characterized in non-intentional terms because intentionality is not the distinguishing feature between software and hardware. Both software and hardware are designed by humans with specific intentions and purposes. Software, characterized by its abstract nature and consisting of instructions and algorithms, manipulates data, while hardware, the physical component, executes these instructions. Although software may seem more directly associated with intentionality, it is ultimately defined by the algorithms it implements, which, without human interpretation, do not necessarily reference external information or meaning. This contrasts with Wakefield’s case of the gosling and the imprinting of the fox’s representation. Wakefield claims that this is a mental dysfunction, as opposed to a brain dysfunction, because it requires referencing the semantic content of the representation encoded in the brain. However, in the case of software and hardware, both can either be described in intentional terms or need not be, underscoring the analogy’s inability to mandate a strict separation between mental and brain dysfunctions.

### Multiple realizability of the mental

Another reason for skepticism about the possibility of reducing mental dysfunction to brain dysfunction relates to the concept of multiple realizability of mental states [11, 38]. Multiple realizability of the mental suggests that the same mental state or function can be realized by different physical states or processes across different individuals or species [39]. This implies that mental states are not tied to a specific neural configuration, making it difficult to pinpoint a one-to-one correspondence between mental and brain dysfunctions. Thus, the variability in how mental functions are instantiated across different brains and bodies might seem to challenge the view that mental dysfunction can be reduced to brain dysfunction.

Even though many seem to subscribe to it, the plausibility of this argument for negating the possibility of reducing mental dysfunction to brain dysfunction has been questioned [for critical discussion, see 15, sec. 8.2]. Specifically, while a mental state/process might not directly reduce to a single brain state/process, it doesn’t follow that a mental dysfunction is independent of brain dysfunction. Indeed, if the underlying brain state/process was selected to perform the relevant mental function, dysfunction in the mental state/process would indicate a dysfunction in the corresponding brain state/process.

Moreover, and more relevant to our context, even if the mental is multiply realizable, it remains unclear why this putative fact would imply that certain brain processes should not be characterized or identified in intentional terms. Nothing in the idea of multiple realizability precludes characterizing the brain with intentional descriptions and applying psychological predicates to it.

So, what considerations might justify the view that brain dysfunction should be characterized in non-intentional terms? We propose that a less-explored line of reasoning, based on an autonomist perspective on the relationship between personal and subpersonal levels of explanation, could, if plausible, justify this view. Authors such as Wakefield [8], Szasz [36], Graham [6] and others may implicitly presuppose this perspective in their views on the nature of mental disorder. In the next two sections, we will examine this option.

## Brain dysfunctions and the distinction between the personal and the subpersonal

While there is still no unitary explanation of the difference, the distinction between the personal and the subpersonal is typically understood as the distinction between explanations that target phenomena at the level of whole agents and subpersonal explanations that target phenomena characterizing processes at the level of parts and components of agents (for recent discussion, see [41–43]). For instance, paradigmatic personal explanations involve the ascription of beliefs, desires, intentions, personality traits, and emotional states to agents, building explanations that utilize those constructs. Paradigmatic subpersonal explanations are found in cognitive (neuro) science, where, for instance, mental operations are associated with neurobiological processes that underlie different psychological functions, such as when dopamine and serotonin neurotransmitters are invoked in explanations of mood changes [44]. If the personal level involves explanations of cognitive phenomena characterizing human agents, while the subpersonal level characterizes neural and other bodily states, then this might potentially ground a principled way for distinguishing mental from brain dysfunction. Mental functions would pertain to cognitive processes that are intentionally specified, while brain functions would be described at the subpersonal level in terms of neurobiological processes.^7^ How this idea might be spelled out in more detail will be addressed in the next subsection.

### Autonomist approaches to the interface problem

To see how the view that brain disorders/dysfunctions need to be determined in structural or physiological terms could be justified by referencing the personal/subpersonal distinction, it is useful to situate the discussion within the interface problem in cognitive science. The interface problem is the problem of explaining the relationship between personal and subpersonal levels of explanation [2, 21]. As mentioned, the personal level is supposed to capture the functioning of agents as such, while the subpersonal level captures explanations of agents in terms of their components [41, 42]. For instance, Daniel Dennett [45] originally introduced this distinction with reference to the case of pain. Pain is normally ascribed to people, not their body parts. Specifically, only people can properly be said to feel pain and behave in certain ways because of feeling pain. Parts of agents, such as their brains, don’t feel pain; thus, anything below the level of the agent seems to be an incorrect target for our concept of pain. Of course, we can discuss the neurobiological processes involving afferent and efferent neural pathways that underlie or are somehow associated with painful experiences. But in such cases, our explanations don’t refer to pain itself; rather, they provide subpersonal explanations of processes occurring in the relevant parts of the agent. The interface problem involves the question of the relationship between personal and subpersonal explanations of psychological phenomena so understood.

There is an influential line of thinking suggesting that similar considerations apply to all psychological predicates, especially those referring to beliefs, desires, and other intentional mental states [24, 46]. Such a view is sometimes called the autonomist view of the personal/subpersonal distinction [21]. This is because these views claim that the personal level forms an explanatorily autonomous domain that is independent from subpersonal states and processes.

According to autonomist views, personal level explanations do not need to be validated by subpersonal discoveries about how the brain functions. The central idea is that mental states properly characterize agents at the personal level of description. Such views often link the personal level with commonsense psychological explanations, which are fundamentally grounded in rational explanations [22, 23]. These explanations depict agents as conscious beings who act based on available reasons, where these reasons refer to the agent’s contentful states, such as those representing goals and beliefs about the best ways to achieve those goals.

In contrast, subpersonal explanations are typically understood in opposition to personal explanations [42]. These explanations address phenomena that exist outside conscious experience and typically deal with parts of agents whose functioning is explained in terms of causal, nomological, or mechanistic factors. As such, these explanations are thought not to apply to agents who act based on reasons; instead, they pertain to components of agents, such as their brains, which can be explained in purely causal-mechanistic terms [22, 46].

There is some indication that Wakefield presupposes such a view when he claims that the distinction between the mental and the physical

“(…) depends on the kinds of concepts essential to describing the function that is failing and the reason for the failure. Cartesian ontological worries aside, brain descriptions and mental content/representational descriptions of the intentional field form *two theoretical domains* with their own functions and lawful relations.” [8] p. 4, emphasis added].

This way of thinking aligns with the autonomist view, which holds that the “intentional field” belongs to the personal domain, while brain descriptions belong to the subpersonal domain. In other words, personal level represents the domain where meaning and intentionality can be appropriately ascribed, whereas the subpersonal level pertains to purely causal mechanisms [22–24].^8^

According to autonomists, the demarcating line between the two domains typically rests on rationality. Attributing contentful or intentional states to agents presupposes a minimal level of rationality [47], as purely causal or non-rational explanations wouldn’t allow us to understand intentional states, like beliefs and desires, as coherent and connected to an agent’s actions. For these states to make sense, they must be interrelated in a way that reflects their rationality—i.e., an agent’s beliefs and desires should be internally coherent, enabling us to understand how actions lead to successful goal attainment. Without this underlying rational structure, the attribution of intentional states would fail because we wouldn’t be able to discern meaningful patterns in agents’ thoughts and behavior. Thus, if subpersonal explanations, which operate at levels below the personal, including neurobiological explanations typically seen as purely causal or mechanistic—and consequently non-rational—are considered, it follows that such subpersonal explanations cannot justify the attribution of intentionality to subpersonal states.

### Autonomism about the personal and the non-intentionality of brain dysfunction

Assuming this autonomist perspective on the relationship between the personal and subpersonal levels of explanation would help explain why brain disorders might not be characterized in intentional terms. Since the brain is a component of an agent, its explanation should be framed in subpersonal terms. At the subpersonal level, explanations are provided in causal or mechanistic terms that do not presuppose rational relations. Thus, descriptions of brain functions can only be articulated in non-intentional terms that refer to the structural and physiological properties of the brain. As a result, brain dysfunctions would be those related to its structural and physiological properties. If such a view is compelling, it would support the claim that mental dysfunctions could be independent of brain dysfunctions. Specifically, mental dysfunctions, as opposed to brain dysfunctions, would involve disturbances at the representational or intentional level of description, which characterize agents at the personal level of functioning.

However, we contend that the autonomist view of the relationship between the personal and the subpersonal is inadequate in the context of psychiatry for the following interrelated reasons. First, it does not correspond to how these notions are used in contemporary (neuro) cognitive sciences and their application to psychiatric disorders. Second, it ignores one of the main reasons for introducing the distinction in the philosophy of cognitive science, namely, to legitimize and make sense of the application of psychological predicates to subpersonal levels of description. Third, such a view does not satisfy one of the requirements for explicating the distinction between the personal and the subpersonal, which pertains to explaining what differentiates subpersonal from nonpersonal explanations. Let us elaborate on these objections in turn.

## The inadequacy of the autonomist view for psychiatry

### Autonomism and the current practice in the sciences of the mind/brain

The autonomist perspective on the distinction between personal and subpersonal explanations is at odds with how the distinction is currently used in cognitive (neuro)science and its application to psychiatry. Cognitive (neuro)scientists typically use the distinction between the personal and the subpersonal to disambiguate at which level of functioning they apply cognitive constructs, including beliefs, utilities, representations, and so on. This is especially evident in the Bayesian brain paradigm and predictive processing accounts of the mind/brain, according to which the brain is understood as an inference machine that, based on incoming stimuli, creates a probabilistic model of environmental causes by performing computations approximating Bayesian inference [48–50]. Here, it is often said that the brain possesses beliefs that form models of the external environment, which it uses to make inferences about what to expect to perceive [51]. However, it is clear that when neurocognitive researchers in this paradigm ascribe beliefs, models, and other cognitive constructs to the brain, they do not have in mind propositional attitudes that we normally apply to people [52]. Instead, they are talking about brain states that encode probability distributions, which may play the role of beliefs, desires, and other cognitive constructs. The distinction between the personal and the subpersonal is often used in this context to differentiate the level at which cognitive constructs are being applied (e.g. psychological, brain networks, neurons) or the type of cognitive construct being used [53–55].

Crucially, even though subpersonal cognitive constructs are typically distinguished from their personal level counterparts, they are still understood as involving information or content that is processed by neural and bodily mechanisms [56]. In this respect, brain functions and structures are endowed with representational and information processing abilities [57]. Importantly, this ascription of representational content is not merely a descriptive label but plays an explanatory role in how these subpersonal states influence behavior [73]. For instance, in the Bayesian brain framework, the brain can be ascribed probabilistic models that represent potential threats in the environment, such as deciding whether a rustling in a nearby bush indicates a dangerous animal, which can guide behavior by preparing a fight-or-flight response based on the predicted level of threat. Thus, typical descriptions of brain functioning go beyond mere anatomical structures or physiological processes. In fact, the use of subpersonal beliefs and inferences in Bayesian brain and predictive processing paradigms transcends the strict separation between the personal and subpersonal levels, thereby dissolving the dichotomy between intentional/mental and non-intentional/physical by allowing for intentional characterizations of brain states [53–55].

This claim becomes even clearer in the emerging field of computational neuropsychiatry, where Bayesian accounts of brain function are applied to explaining different aspects of mental disorders [51]. For instance, some studies indicate that delusions and psychotic symptoms associated with schizophrenia can be explained by aberrant processing of belief revision in response to how uncertainty is represented within the brains of patients with respect to incoming stimuli [58–60]. Thus, the practice of cognitive neuroscientists and neuropsychiatrists does not support the view that subpersonal explanations should only refer to non-intentionally described brain processes [50].

It might be objected that the use of cognitive constructs in cognitive neuroscience is metaphorical and should, at most, be given an instrumental reading [24]. If ascriptions of brain dysfunction involving some form of intentionality are merely metaphorical, because the brain does not truly engage in intentional processes that provide information with semantic content, then there could not really be dysfunctional brain processes.

Now, the question arises: what could warrant such a view? The answer cannot simply be that only the personal level warrants ascriptions of psychological predicates and their counterparts, as this would beg the question against those who argue that there are sufficient reasons to apply such predicates even to subpersonal processes [61–63, 73]. A more constructive approach to advancing this problem is to ask: what would provide sufficient reasons for ascribing psychological predicates to any system under consideration? Answering this question will lead us to the second reason for questioning the autonomist view of relation between the personal and the subpersonal.

### When is it appropriate to ascribe intentional properties to the brain?

In her influential discussion of the problem of understanding the notions of the personal and the subpersonal, Zoe Drayson [61] proposes that the distinction’s primary purpose was to legitimize the widespread practice in cognitive sciences of ascribing psychological predicates to parts of persons. How is this practice justified? Typically, the justification for introducing psychological descriptions is instrumental. If the system under consideration is sufficiently similar in relevant respects to paradigmatic cognitive systems, i.e., rational agents, and this similarity provides explanatory and epistemic benefits, then we are justified in applying the relevant psychological predicates to those systems [64, 73].

In contemporary cognitive sciences, this practice is often noticeable through the application of scientific models. For instance, if a scientific model of one phenomenon can successfully be applied to another phenomenon, it provides evidence that the processes described are similar according to the main parameters of the model [for discussion, see [62]). This is typically how psychological predicates are transferred from personal descriptions to subpersonal processes. More specifically, this is how the idea of the Bayesian brain was developed in many of its facets [65]. Across several studies, it has been shown that economic Bayesian models of decision-making, typically used for different aspects of rational agency, have been successfully applied to predict data related to how the brain processes perceptual information and produces motor signals [66]. From the successful application of these models, many researchers have concluded that it is meaningful to apply psychological predicates, such as representations, preferences, and utilities, which typically characterize whole cognitive agents, to subpersonal brain processes [52, 67, 68, cf. 69].

However, finding that a scientific model of one phenomenon applies to another phenomenon is not sufficient to warrant the claim that all properties of the original system the model was intended to represent can be transferred to the properties of the newly modeled system. For instance, it would not be compelling to claim that if a model created to represent certain aspects of fluid dynamics can be used to predict traffic dynamics, we can conclude that traffic is a kind of fluid and possesses all the properties of fluids [70]. Therefore, to justify the thinking that the successful application of a model from one domain to another implies that these two domains are, in relevant respects, the same, there must be reasons not solely connected to the model.

In the case of psychological abilities and the feasibility of characterizing the brain in Bayesian terms, such reasons are readily available. On naturalistic views, the mind consists of capacities enabled by the brain, and the mind is shaped by natural processes that also shape the brain to enable the organism to survive and prosper in its environment [28]. Thus, if the relevant properties of certain mental capacities can be represented by models that informatively and explanatorily fit how some parts of the brain function, given the intimate connection between mental and brain capacities, this would warrant ascribing the relevant mental capacity to the brain or even thinking that we have identified a neural realization of that capacity [61, 62].

### Distinguishing the subpersonal from the nonpersonal

The third reason why the autonomist view of the relationship between the personal and subpersonal is not the most compelling, at least in the context of psychiatry, is that it appears to dismiss the relevance of the subpersonal by reducing it to the nonpersonal [42]. On the face of it, those who claim that only personal processes are intentional, and that all subpersonal processes are non-intentional, face the problem of explaining the “sub” in *sub*personal and what distinguishes such processes from nonpersonal ones (for recent discussion, see [41, 43]). We maintain that an adequate account of personal and subpersonal explanations should provide an answer to this question.

However, the most perspicuous explanation of the difference between subpersonal and nonpersonal explanations, at least as used in prominent discussions, is that subpersonal explanations refer to the cognitive capacities of subagents, i.e., parts and processes of whole agents, while nonpersonal explanations do not pertain specifically to cognitive functions or abilities, whether of agents or other objects [61]. In this regard, descriptions of brain processes that implement predictive inferences of incoming stimuli and adjust motor actions to adaptively respond to environmental challenges would involve standard subpersonal explanations. In contrast, the description of how human skin absorbs water via osmosis would be a paradigmatic nonpersonal explanation, as no cognitive process is involved. Given that at least some brain processes can be described as involving representation and other intentional phenomena, they can be characterized in subpersonal terms and should be distinguished from other processes in the brain that are nonpersonal, such as those involving osmosis in different parts of the body.

From this perspective, it becomes clear that we have another reason to reject Wakefield’s [8] and similar views that brain disorders should be characterized in non-intentional terms. If there are legitimate cases of subpersonal processes that characterize brain function, then these processes could also malfunction. These would be cases in which some brain dysfunction would not be identifiable in purely anatomical or physiological terms, as its function and dysfunction are determined by how well it performs some cognitive task. For instance, in the Bayesian brain framework, delusions can be understood as a result of a mismatch in how the brain processes top-down predictions and bottom-up sensory information [58]. The idea is that, in normal cases, the brain uses predictive models to anticipate incoming sensory information, and when there’s a mismatch, it updates its predictions to better fit the sensory input. However, in schizophrenia, there might be an imbalance or disruption that induces irrationality in predictive processing [53], as individuals with schizophrenia may have overly strong top-down predictions that are not adequately corrected by bottom-up sensory information [59, 60]. This could lead to persistent beliefs or delusions that are not aligned with the actual state of affairs, as the brain fails to update its predictions efficiently. In such cases, where brain dysfunction is described in terms of inferential processes, adopting Wakefield’s view would obscure those ways in which the brain can malfunction at the subpersonal level of processing.

## Conclusion: Limitations and future directions

In this paper, we have examined a less-discussed reason for considering mental disorders as autonomous from brain disorders, specifically that mental dysfunctions can be understood as independent of brain dysfunctions. We have focused on the argument according to which many mental disorders are defined by dysfunctional *intentional* processes, while brain dysfunctions are defined by anatomical or physiological abnormalities that cannot be described in psychological/intentional terms [6, 8, 36].

This paper examined several ways in which this presupposition about the independent non-intentional specifiability of brain dysfunction could be defended. More specifically, we traced a potential justification of this view in the autonomist perspective on the personal and subpersonal levels of explanation. However, we argued that this view is incompatible with how the distinction is employed in cognitive science and (neuro)psychiatry. Additionally, we argued that it overlooks the key philosophical rationale for the distinction, which is to justify the application of psychological predicates to subpersonal levels. Finally, we argued that it fails to clarify the crucial distinction between subpersonal and nonpersonal explanations, which is essential for differentiating the two.

A limitation in the current discussion should be noted. Our primary aim was to examine why brain dysfunctions might be considered independent of mental dysfunctions, with our analysis situated within the mainstream view that mental states minimally supervene on brain states. However, this leaves open how the discussion might evolve if alternative conceptions of the mind and brain were adopted. For example, our conclusions might require modification under the extended mind hypothesis, according to which the mind extends beyond the brain to include external artifacts and processes, such as pencils, notebooks, or electronic devices [33]. Future studies could explore this possibility in greater depth.

Nonetheless, we think that even if the extended mind view is assumed, it would not undermine our main argument against the view that brain dysfunctions should be characterized solely in non-intentional terms. This is because the extension of the mind to artifacts outside the brain and body does not imply anything about how the part of the mind that supervenes on the brain and other bodily processes should be characterized. Thus, we conclude that even under this supposition, there is no sufficient reason to believe that mental dysfunctions cannot, in principle, be reduced to brain dysfunctions.

## Data Availability

No datasets were generated or analysed during the current study.

## Footnotes

1 It should be noted that the relationship between dysfunction, disorder, and related terms such as pathology and illness has been approached in various ways within the literature [see 16, ch. 2]. In particular, some theorists equate disorder with dysfunction, while others distinguish between them [17, 18]. Among those who distinguish between dysfunction and disorder, the most influential account is Jerome Wakefield’s [19] hybrid view according to which mental disorders are negatively evaluated conditions that are caused by a dysfunctional psychological mechanism. Wakefield’s view will be further elaborated in Sect. 2. This paper proceeds on the assumption that, at a minimum, a hybrid view is correct, which suggests that mental disorder involves dysfunction as a necessary condition while leaving open the possibility that an additional criterion may be required for a full account [see, also 20].

2 Wakefield [8, 26] discusses this issue in the context of whether addiction should be considered a medical disorder. Given that his particular case study is not relevant to our discussion, we will not reference it in what follows. For recent discussion of the problem of addiction as a disorder and how it influences moral responsibility judgments, see, e.g., [27].

3 Indeed, as a reviewer of this paper pointed out, many would firmly contend that this example illustrates merely a mismatch between the imprinting system and the external environment, rather than a genuine case of dysfunction [e.g. 29, ch. 8, 71, 72].

4 Similar views to Wakefield’s on the idea that brain disorders should be characterized in terms of anatomical and physiological abnormalities are most notably endorsed by Thomas Szasz [36] and, more recently, by George Graham [6]. Anneli Jefferson [11, 40].

5 According to etiological theories of biological function/dysfunction, a trait’s function is determined by its history of natural selection [29]. Dysfunction occurs when a trait fails to perform the role it was selected to do [19]. For example, the function of the reproductive system is to enable pregnancy and successful reproduction because these roles have been selected for through evolution. If there is an issue, such as infertility or recurrent miscarriages, where the reproductive system cannot sustain a pregnancy, it is considered dysfunctional. (The example with pregnancy was chosen deliberately, as issues related to pregnancy are too often neglected in philosophical work, see [32].).

6 The argument becomes more complicated if we consider the extended view of the mind, which argues that external physical items and processes can be constitutive parts of the mind [33]. In that case, if some mental functions depend on external features of the environment, not all functions of the mind could be reduced to the designed functions of the brain. Whether this poses a real threat to Fagerberg’s [15] argument is a topic for another discussion, as it is not the primary issue addressed in this paper. Thanks to Fabian Hundertmark for highlighting this potential issue.

7 It should be noted here that this way of presenting the distinction between the personal and the subpersonal—where the subpersonal is paradigmatically associated with neural processes—is common across different views on the personal/subpersonal relationship [21]. However, this should not create the impression that cognitive sciences do not recognize subpersonal states described outside the level of neural processes. In fact, in Sect. 5, we will argue that it’s common practice in cognitive sciences [see, e.g. 73] to presuppose that subpersonal states can be described in intentional terms, which poses a challenge for those who claim that brain states/processes should be described solely in non-intentional terms. I thank the reviewer for highlighting the need to make this point more explicit.

8 For an extended discussion of Szasz’s [36] subscription to a similar autonomist view, see [40].
